# Mother–Infant HIV Transmission: Do Maternal HIV-Specific Antibodies Protect the Infant?

**DOI:** 10.1371/journal.ppat.1004283

**Published:** 2014-08-21

**Authors:** Julie Overbaugh

**Affiliations:** Division of Human Biology, Fred Hutchinson Cancer Research Center, Seattle, Washington, United States of America; University of Florida, United States of America

## Mother-to-Child Transmission (MTCT) of HIV—The Basics

Despite the intimate relationship between a mother and her fetus, the majority of HIV-infected pregnant women do not transmit HIV to their infant. Approximately one-third of exposed infants acquire HIV in the absence of any preventative interventions, with exposure during delivery and breastfeeding accounting for the majority of infections; in utero infections contribute a relatively small fraction [1], [2]. Because of successes with antiretroviral (ARV) treatment to prevent infection, the number of HIV-infected infants is declining, but there were nonetheless approximately a quarter million new infant infections in the past year [3].

HIV is found in blood, genital secretions, and breast milk, with higher levels in each of these body fluids correlated with transmission [1], [4]. Given the strong associations between maternal virus levels and transmission, considerable effort has been placed on reducing maternal viral burden through ARV therapy during pregnancy, delivery, and breastfeeding. This approach, combined with providing infants with ARVs as prophylaxis, can reduce transmission levels to a few percent [5]. Preventing mother-to-child transmission (MTCT) has been a great success story in HIV prevention efforts, although it presents challenges in identifying and treating those at risk and related issues of drug resistance [5]–[7].

In addition to providing key insights into the use of treatment for prevention of HIV transmission, MTCT has also offered insights into the potential of HIV-specific immune responses to provide protection—a topic that is central to rational HIV vaccine design. Much of the focus has been on neutralizing antibodies (Nabs) because the transfer of passive antibodies from mother to infant creates a unique situation in which the infant has HIV-specific Nabs at the time of exposure, much like what would be expected with a vaccine designed to elicit antibodies. Antibodies are transferred across the placenta and reach high levels at the time of birth. Thus, during late gestation and breastfeeding, the infant has HIV-specific antibodies potentially capable of recognizing and neutralizing the maternal virus. The fact that transmission occurs in the face of these passive antibodies suggests that they are not highly effective at blocking transmission. However, more than 60% of untreated HIV-exposed infants do resist transmission, leaving open the possibility that antibodies are effective in some settings, either when they are present at high enough levels at the place and time of exposure and/or have the proper specificity or function. Studies to address these possibilities have yielded variable results, as discussed below.

## What Role Do HIV-Specific Neutralizing Antibodies Play in Protection?

There is, as yet, no clear picture on how much of a role HIV-specific Nabs play in protection of a HIV-exposed infant, but the weight of evidence seems to suggest they may contribute. Several small studies where Nabs were specifically measured against the autologous maternal virus suggested a partially protective effect of maternal Nabs on transmission [8]–[10]. However, results vary across studies, with a recent study even suggesting an enhancing effect of maternal Nabs on transmission [11]. Moreover, some studies reported that Nabs protect the infant only in utero [10], while others suggested it is only during delivery [12]. To some extent, the variation can be attributed to the small sample sizes of most studies, making it challenging to consistently identify associations. A potentially more problematic variable is the timing of when antibodies and virus were characterized in relation to when transmission occurred in some studies. HIV has a high rate of genetic variation and changes rapidly in response to immune pressures; the host immune response, which is also very dynamic in nature, adapts in kind. This clash of the evolutionary titans [13] means that the study of immune response correlates outside the window when transmission occurred may be largely irrelevant to understanding the role of antibodies in protection. In this regard, it is important to remember that, unlike experimental systems, it is virtually impossible to examine events at the time of infection in humans. Therefore, the ability to address these questions in human studies depends both on how closely the time of infection can be defined and when samples are available in relation to it.

Our studies of larger cohorts of mother–infant pairs near the time of transmission have suggested that neither the breadth of the maternal HIV-specific Nab response nor the breadth of the passively acquired HIV-specific Nabs in the infant correlated with risk of infant infection [14], [15]. The caveat to these studies is that Nab activity was measured against representative HIV variants circulating in the population (heterologous variants), not the individual autologous viruses from each mother–infant pair, as was done in some of the smaller studies. Thus, while the larger studies suggest limited benefit of broadly active Nabs in protection, these findings do not provide a definitive answer as to whether Nabs provide some protection against the specific HIV variants that the infant encounters. Studies using autologous virus are difficult to do on a large enough scale to convincingly address this complex question, but if this were undertaken, it would provide valuable information on the potential of HIV-specific Nabs to protect against HIV infection.

## Do Nabs Select for Transmission of Escape Variants?

**Figure 1:**
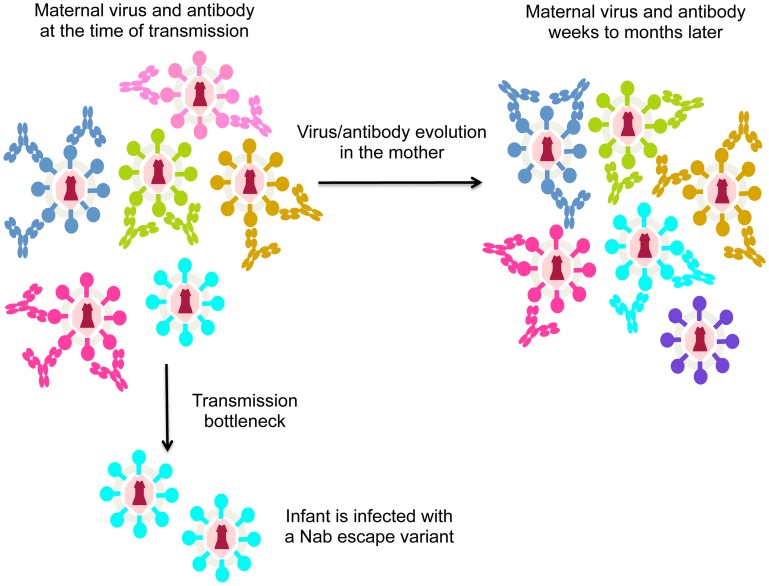
Schematic of virus escape from antibody in MTCT. Different virus variants are shown in different colors, with the antibodies that recognize and neutralize them shown in the same color. For simplicity, in this figure each antibody only recognizes one virus variant, although in reality, some will recognize several different variants. During the process of transmission (transmission bottleneck) the infant is infected with a viral variant from the mother that was not neutralized by her antibodies. The arrow indicating evolution in the mother shows how virus/antibody dynamics change. In particular, the virus that was not recognized by maternal antibodies at the time of transmission (shown in cyan) may be recognized by newly elicited antibodies present a few weeks later. At that time, a different escape variant (shown in purple) may have evolved.

As noted above, HIV is highly variable and rapidly escapes the Nab response [13]. Thus, in the case of MTCT, Nabs may not protect simply because the mother often harbors escape variants that are poorly neutralized by her antibodies. In the case that the mother harbors virus variants with a range of neutralization sensitivities, the virus transmitted to the infant should be one that is poorly recognized by maternal Nabs if Nabs are effective at protecting against neutralization sensitive variants (Figure 1). In fact, there is evidence to support this model. Larger studies focused on virus–antibody dynamics near the window of transmission showed that the viruses transmitted to infants were significantly less sensitive to maternal Nabs than the overall maternal virus population [16], [17]. These findings suggest that Nabs are blocking some HIV variants, but they cannot block the harder-to-neutralize viruses that have undergone escape. To accomplish escape, the variants transmitted to infants appear to have altered their conformation to mask epitopes recognized by maternal Nabs [18]. Of note, recent studies have provided insight into the structure of a trimeric envelope protein representing an escape variant that was transmitted to an infant, including envelope protein in complex with antibody [19]. Studies of this type, particularly those comparing the structure and antibody-binding properties of maternal versus infant variants, could help define the antibody selective pressure that leads to transmission of Nab escape variants in infants.

In aggregate then, studies to-date provide evidence for a role for Nabs in blocking some HIV-1 variants in MTCT. However, the jury is still out on whether they contribute in any significant way to blocking infection completely, and this may be partly due to the rapid evolution of Nab escape variants in the mother (Figure 1).

## What Role Do Antibodies That Mediate Killing of Infected Cells Play in Protection from MTCT?

Interestingly, there is evidence from one recent study that antibodies that act through antibody-dependent cellular cytotoxicity (ADCC) may contribute to infant protection, particularly ADCC antibodies in breast milk [20]. In humans, breast milk does not substantially contribute to passive transfer of circulating antibodies in the infant [21] and thus are not acting in the infant to block virus entry. However, they could play a role in protection by reducing virus levels in breast milk and thus reducing infectiousness. Given that antibodies that act through ADCC have the potential to kill infected cells, the link between breast milk ADCC antibodies and infant infection is particularly interesting because cell-associated virus has been implicated in breast milk HIV transmission [4]. It remains unclear if ADCC antibodies in breast milk contribute to reducing infant infection risk by killing infected cells, or whether they are simply a surrogate marker for ADCC antibodies circulating in the mother or in the infant, as these activities are likely highly correlated. In addition, the data comes from just one relatively small study [20]. The role of ADCC antibodies in MTCT is therefore a topic that needs more exploration, especially given the suggestion that ADCC antibodies may have contributed to protection in a HIV vaccine trial in humans, at least in a subset of individuals [22].

## Summary

MTCT has been a rich source of information for prevention research; it has demonstrated the benefit of using ARVs to block transmission and it has also provided insights into the potential of antibodies to prevent HIV infection. However, understanding the factors that lead to the majority of HIV-exposed infants eluding infection is challenging because the determinants of risk are clearly multifactorial. In that regard, other immune responses may contribute to this protection, including cellular immune mechanisms and innate factors [1], [2]. Studies of immune correlates of protection are also complicated by the dynamic nature of HIV and the immune response to it.

While the current state of knowledge suggests that antibody-mediated protection may not be the major factor in determining if an infant acquires HIV from their mother, it may play a role. There is some provocative but relatively limited evidence that antibodies may protect infants via ADCC. In the case of Nabs, several small studies have shown a correlation between Nabs and protection, but results of studies on this topic are variable and would benefit from larger studies focused specifically on the window of transmission. There is perhaps better evidence that antibodies contribute to blocking virus variants that are highly sensitive to neutralization, suggesting that the Nabs elicited in a typical infection may not have adequate breadth and/or potency to prevent transmission of the harder-to-neutralize viruses. This may be a peculiarity of MTCT, where escape variants elicited specifically to maternal antibodies are often present. MTCT could therefore provide insights on the potency of antibody needed for protection if we can understand which subset of maternal variants are blocked by antibodies and if some mothers have antibodies of sufficient breadth and potency to completely prevent infant infection. Understanding how much antibody is needed to block infant infection could be invaluable in helping guide vaccine design, where the bar for eliciting antibody-based protection in humans is poorly defined.

## References

[1] Lehman DA, Farquhar C (2007) Biological mechanisms of vertical human immunodeficiency virus (HIV-1) transmission. Rev Med Virol 17: 381–403 10.1002/rmv.543 17542053

[2] Tobin NH, Aldrovandi GM (2013) Immunology of pediatric HIV infection. Immunol Rev 254: 143–169 10.1111/imr.12074 23772619 PMC3737605

[3] UNAIDS (2013) Global Report: UNAIDS report on the global AIDS epidemic 2013. Available: http://www.unaids.org/en/media/unaids/contentassets/documents/epidemiology/2013/gr2013/unaids_global_report_2013_en.pdf. Accessed 22 July 2014.

[4] Milligan C, Overbaugh J (2014) The Role of Cell-Associated Virus in Mother-to-Child HIV Transmission. J Infect Diseases In press. 10.1093/infdis/jiu344 PMC4303081 25414417

[5] Chi BH, Stringer JSA, Moodley D (2013) Antiretroviral Drug Regimens to Prevent Mother-To-Child Transmission of HIV: A Review of Scientific, Program, and Policy Advances for Sub-Saharan Africa. Curr HIV/AIDS Rep 10: 124–133 10.1007/s11904-013-0154-z 23440538 PMC3644371

[6] Paredes R, Marconi VC, Abrams EJ, Lockman S, Kuhn L (2013) Impact of Antiretroviral Drugs in Pregnant Women and Their Children in Africa: HIV Resistance and Treatment Outcomes. J Infect Diseases 207 Suppl 2: S93–S100 10.1093/infdis/jit110 23687295 PMC3657116

[7] Lehman DA, John-Stewart GC, Overbaugh J (2009) Antiretroviral strategies to prevent mother-to-child transmission of HIV: striking a balance between efficacy, feasibility, and resistance. PLoS Med 6: e1000169 10.1371/journal.pmed.1000169 19859532 PMC2760781

[8] Scarlatti G, Albert J, Rossi P, Hodara V, Biraghi P, et al (1993) Mother-to-child transmission of human immunodeficiency virus type 1: correlation with neutralizing antibodies against primary isolates. J Infect Dis 168: 207–210. 8515110 10.1093/infdis/168.1.207

[9] Kliks SC, Wara DW, Landers DV, Levy JA (1994) Features of HIV-1 that could influence maternal-child transmission. JAMA 272: 467–474. 8040983

[10] Dickover R, Garratty E, Yusim K, Miller C, Korber B, et al (2006) Role of maternal autologous neutralizing antibody in selective perinatal transmission of human immunodeficiency virus type 1 escape variants. J Virol 80: 6525–6533 10.1128/JVI.02658-05 16775339 PMC1488973

[11] Baan E, De Ronde A, Stax M, Sanders RW, Luchters S, et al (2013) HIV-1 Autologous Antibody Neutralization Associates with Mother to Child Transmission. PLoS ONE 8: e69274 10.1371/journal.pone.0069274.t002 23874931 PMC3714266

[12] Barin F, Jourdain G, Brunet S, Ngo-Giang-Huong N, Weerawatgoompa S, et al (2006) Revisiting the role of neutralizing antibodies in mother-to-child transmission of HIV-1. J Infect Dis 193: 1504–1511 10.1086/503778 16652277

[13] Burton DR, Stanfield RL, Wilson IA (2005) Antibody vs. HIV in a clash of evolutionary titans. Proc Natl Acad Sci U S A 102: 14943–14948 10.1073/pnas.0505126102 16219699 PMC1257708

[14] Lynch JB, Nduati R, Blish CA, Richardson BA, Mabuka JM, et al (2011) The breadth and potency of passively acquired human immunodeficiency virus type 1-specific neutralizing antibodies do not correlate with the risk of infant infection. J Virol 85: 5252–5261 10.1128/JVI.02216-10 21411521 PMC3094986

[15] Omenda MM, Milligan C, Odem-Davis K, Nduati R, Richardson B, et al (2013) Evidence for Efficient Vertical Transfer of Maternal HIV-1 Envelope-Specific Neutralizing Antibodies but No Association of Such Antibodies with Reduced Infant Infection. J Acquir Immune Defic Syndr 64: 163–166 10.1097/QAI.0b013e31829f6e41 23774880 PMC3805370

[16] Wu X, Parast AB, Richardson BA, Nduati R, John-Stewart G, et al (2006) Neutralization escape variants of human immunodeficiency virus type 1 are transmitted from mother to infant. J Virol 80: 835–844 10.1128/JVI.80.2.835-844.2006 16378985 PMC1346878

[17] Zhang H, Rola M, West JT, Tully DC, Kubis P, et al (2010) Functional properties of the HIV-1 subtype C envelope glycoprotein associated with mother-to-child transmission. Virology 400: 164–174 10.1016/j.virol.2009.12.019 20096914 PMC2844456

[18] Goo L, Milligan C, Simonich CA, Nduati R, Overbaugh J (2012) Neutralizing Antibody Escape during HIV-1 Mother-to-Child Transmission Involves Conformational Masking of Distal Epitopes in Envelope. J Virol 86: 9566–9582 10.1128/JVI.00953-12 22740394 PMC3446598

[19] Julien J-P, Cupo A, Sok D, Stanfield RL, Lyumkis D, et al (2013) Crystal structure of a soluble cleaved HIV-1 envelope trimer. Science 342: 1477–1483 10.1126/science.1245625 24179159 PMC3886632

[20] Mabuka J, Nduati R, Odem-Davis K, Peterson D, Overbaugh J (2012) HIV-specific antibodies capable of ADCC are common in breastmilk and are associated with reduced risk of transmission in women with high viral loads. PLoS Pathog 8: e1002739 10.1371/journal.ppat.1002739 22719248 PMC3375288

[21] Van de Perre P (2003) Transfer of antibody via mother's milk. Vaccine 21: 3374–3376. 12850343 10.1016/s0264-410x(03)00336-0

[22] Haynes BF, Gilbert PB, McElrath MJ, Zolla-Pazner S, Tomaras GD, et al (2012) Immune-correlates analysis of an HIV-1 vaccine efficacy trial. N Engl J Med 366: 1275–1286 10.1056/NEJMoa1113425 22475592 PMC3371689

